# Infection Risk in Dermatology Patients Receiving Next-Generation Medication: A Meta-Analysis of JAK Inhibitors and Biologics

**DOI:** 10.3390/medicina61112053

**Published:** 2025-11-18

**Authors:** Aditya K. Gupta, Susmita, Vasiliki Economopoulos

**Affiliations:** 1 Division of Dermatology, Department of Medicine, Temerty Faculty of Medicine, University of Toronto, Toronto, ON M5S 1A8, Canada; 2 Mediprobe Research Inc., London, ON N5X 2P1, Canada; ssusmita@mediproberesearch.com (S.); veconomopoulos@mediproberesearch.com (V.E.)

**Keywords:** biologics, JAK inhibitors, infection, psoriasis, atopic dermatitis

## Abstract

*Background/Objectives*: Next-generation drugs, such as JAK inhibitors and biologics, have proved to be very effective treatment choices in several autoimmune and autoinflammatory skin disorders. However, these drugs are not without risk. Due to their immune-modulating properties, these drugs may pose a risk of infection, which could vary between drug target, disorder type and pathogen. Our goal was to determine infection risk and how it may vary by drug target, pathogen and skin disorder, namely psoriasis, atopic dermatitis, alopecia areata, vitiligo and hidradenitis suppurativa. *Methods*: We performed a systematic search and meta-analysis where we extracted the rates of different infections from the adverse events of each trial that were found and met our inclusion criteria. *Results*: We found significant associations in psoriasis and atopic dermatitis where infection risk varied by drug, skin condition and pathogen type. We specifically found that there was an increased risk of viral infection for patients with atopic dermatitis with both JAK inhibitors and biologics. We also found an increased risk of fungal infections in psoriasis patients receiving targeted therapies. Lastly, we observed a decreased risk of bacterial infections in atopic dermatitis with dupilumab specifically. Additionally, there was a significantly higher incidence of herpes simplex infections in atopic dermatitis patients with target-selective JAK inhibitors, while no increased risk was observed with herpes zoster. *Conclusions*: There is a varied risk with these next-generation medications that needs to be considered when determining treatment regime.

## 1. Introduction

Skin disorders are some of the most noticeable afflictions that a person can face. These disorders, which include psoriasis (PsO), alopecia areata (AA), atopic dermatitis (AD), hidradenitis suppurativa (HS) and vitiligo, can have a profound effect on a patient’s well-being [1,2,3,4,5].

Next-generation immunomodulating drugs, such as janus kinase inhibitors (JAKis) and biologics including monoclonal antibodies (mAbs), are an effective treatment option that has revolutionized the treatment of autoimmune and autoinflammatory skin disor-ders [6,7]. These medications have proved to be effective options in treating these disorders, with a plethora of clinical trials in AD and PsO in particular.

However, with the increased use of these drugs, we need to have a detailed understanding of their safety, particularly the risk of infection. From older disease-modifying anti-rheumatic drugs (DMARDs), which include oral corticosteroids, methotrexate and cyclosporin, we know that there is an increased risk of infection [8,9].

As these newer drugs more specifically target inflammatory pathways that are associated with viral, bacterial and fungal immunity, there is the possibility that some of these drugs may make patients more susceptible to certain infections. It has been previously shown that JAKi are associated with an increased risk of varicella zoster infections, but not with serious infections [10]. Additionally, Yiu et al. examined infection risk with biologics in PsO patients, where no significant difference was found. We believe that specific targeting of immunological pathways within AD, PsO, HS, AA and vitiligo may leads to an increased risk of infections that differs between disorders and drug classes/targets. Our goal was to perform a meta-analysis to determine the associated risk of fungal, bacterial and viral infection for each drug class/target and disorder, making this study unique compared to previously published work. We have also used a general linear mixed model (GLMM) to perform this study which is another unique aspect of this work.

## 2. Materials and Methods

**Figure 1:**
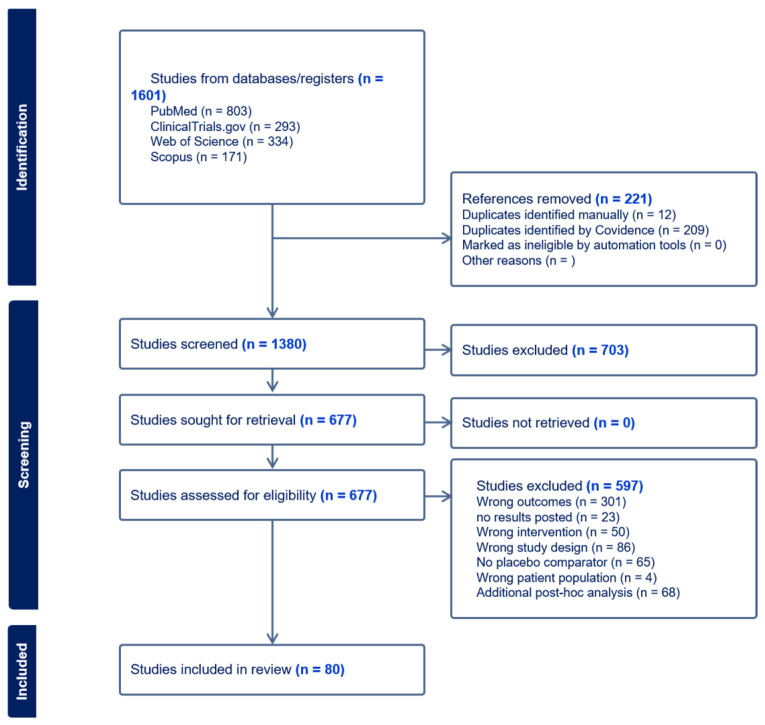
PRISMA chart of systematic search.

We performed a systematic search for published clinical trials using Pubmed, Scopus, Web of Science and ClinicalTrials.gov on 7 August 2025 and registered this study with PROSPERO (CRD420251149970). The details of our search strings and inclusion/exclusion criteria can be found in Tables S1 and S2. Briefly, we searched these databases for all relevant articles of clinical trials of biologic medications and JAKi in patients with AD, PsO, AA, vitiligo or HS, and provided bacterial, viral or fungal infection rates. The Covidence software platform (https://www.covidence.org/, accessed on 7 August 2025; Australia) was used to manage title and abstract screening, full text review and data extraction. The PRISMA chart for this work can be found within Figure 1. Any discrepancies that occurred were assessed by an independent reviewer.

Using the collected data, we conducted a meta-analysis of proportions to examine infection risk between drug and placebo trial arms. We used a general linear mixed model (GLMM) to perform our analysis, using a binomial distribution and a logit link function. The GLMM is preferable as proportional data tends to follow a binomial distribution, which we can specify within the model, whereas traditional meta-analysis methods assume a normal distribution.

All of our analyses were performed using SAS Studio 3.82 (Statistical Analysis Software, Inc., Cary, NC, USA). We used a significance level of a = 0.05 for all comparisons with multiple comparison adjustments (Dunnett–Hsu) made to *p*-values where appropriate.

N-values and heterogeneity (*I*^2^) statistics for each comparison are shown in Table 1. Doi plots to assess publication bias are shown in Figure S1. We have presented all proportions as mean (95% confidence interval) and have presented all odds ratios (ORs) as OR (95% confidence interval). In Table S3, we have provided a summary of all the studies and trials included within this analysis. In Table S4, we have provided the risk of bias assessment for this study.

## 3. Results

We performed our meta-analyses using data extracted from 80 individual trials (Figure 1 and Table S3), where we analyzed the rates of infections for fungal, bacterial and viral infections in JAKi and biologics. We additionally analyzed the rates of herpes simplex infections.

### 3.1. Study Characteristics

The majority of studies in this analysis evaluated adverse events for each drug at timepoints of either 12 or 16 weeks (Table S3); however, some studies have longer evaluation timepoints of 24 or 36 weeks. Only one study evaluated adverse events for up to 200 weeks.

In Table 1 above, we have displayed the number of patients and the heterogeneity statistic for each analysis that was conducted. We have taken a heterogeneity of 0 to less than 30 to be low heterogeneity, 30 to less than 50 to be moderate heterogeneity and 50 and above to be high heterogeneity. The majority of our analyses had low to moderate heterogeneity. We found high heterogeneity in our analysis of herpes simplex and herpes zoster within PsO, and for viral infections within HS.

We also conducted a risk of bias (RoB-2 tool), which is shown in Table S4. We found that the vast majority (74 of 80) of the included studies had a low risk of bias.

### 3.2. Fungal Infections

**Figure 2:**
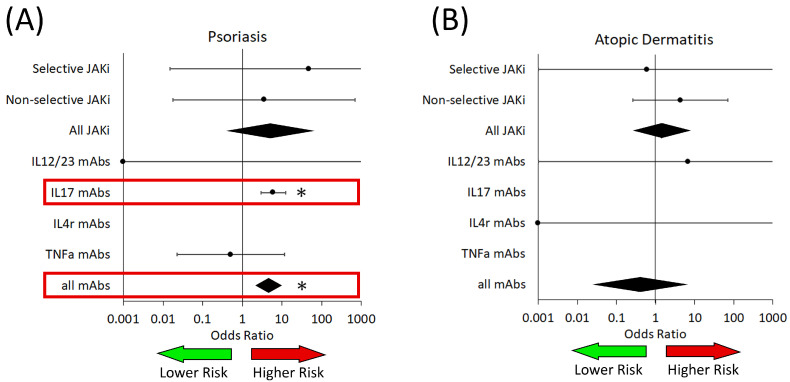
Risk of fungal infection in patients from analyzed trials of both JAKi and biologic medications (mAbs). (**A**) Patients with psoriasis. (**B**) Patients with atopic dermatitis. IL—interleukin; TNFa—tumor necrosis factor alpha. The green arrow represents the area on the forest plot of reduced risk, while the red arrow represents the area of increased risk. Red boxes highlight significant associations with increased risk. Black diamonds are ORs for all JAK inhibitors and all biologics. * *p* < 0.05.

When we analyzed the rates of fungal infections in patients with PsO, we found that no associations were found for JAKi (Figure 2 and Table 2) or their subgroups (Table 3). We found that patients who received any biologic medication had a significantly increased risk of fungal infections (OR: 4.53 [2.34–8.78]). More specifically, through subgroup analysis, we found that -targeted biologics (Table 4) were associated with an increased risk (OR: 6.07 [2.96–12.44]).

We also examined fungal infection risk in AD, AA, and HS, where we did not observe any differences in infection rates compared to placebo in any studied drug. There were no trials that reported fungal infections within vitiligo.

### 3.3. Bacterial Infections

**Figure 3:**
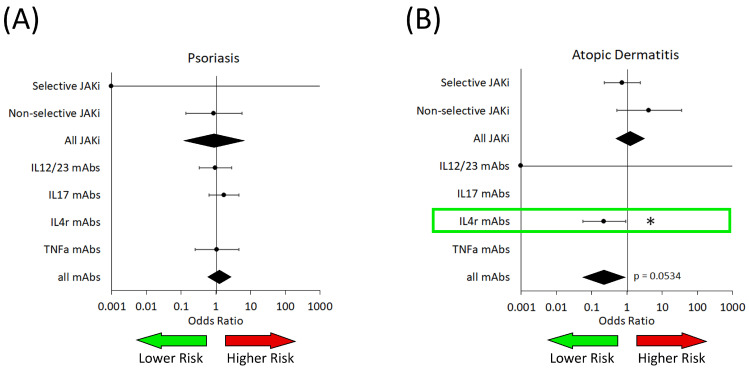
Risk of bacterial infection in patients from analyzed trials of both JAKi and biologic medications (mAbs). (**A**) Patients with psoriasis. (**B**) Patients with atopic dermatitis. IL—interleukin; TNFa—tumor necrosis factor alpha. The green arrow represents the area on the forest plot of reduced risk, while the red arrow represents the area of increased risk. The green box highlights a significant association with decreased risk. Black diamonds are ORs for all JAK inhibitors and all biologics. * *p* < 0.05.

We analyzed the associated infection risk in patients. We found that in PsO patients, there were no significant differences observed for any drug target. However, in AD patients, we found that IL-4R-targeted biologics—dupilumab—were associated with a decreased risk of bacterial infection (OR: 0.23 [0.06–0.92]) (Figure 3 and Table 4).

There was no difference observed in bacterial infection risk in patients with HS who received a biologic. We did not have enough trial data to calculate a result for either class of drugs in AA, while there was no data available for bacterial infections in patients with vitiligo.

### 3.4. Viral Infections

**Figure 4:**
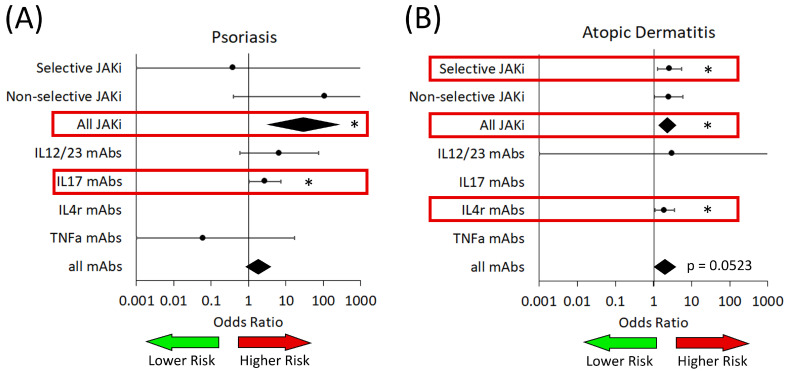
Risk of viral infection in patients from analyzed trials of both JAKi and biologic medications (mAbs). (**A**) Patients with psoriasis. (**B**) Patients with atopic dermatitis. IL—interleukin; TNFa—tumor necrosis factor alpha. The green arrow represents the area on the forest plot of reduced risk, while the red arrow represents the area of increased risk. Red boxes highlight significant associations of increased risk. Black diamonds are ORs for all JAK inhibitors and all biologics. * *p* < 0.05.

Examining the rates of viral infections, we find that there are increased risks for some drugs (Figure 4). In patients with PsO, we found a significant risk of viral infections in patients who received any type of JAKi (OR: 29.08 [3.06–276.36]) (Table 2). In our subgroup analysis, patients who received IL-17-targeted biologics had a significantly increased risk of viral infection (OR: 2.72 [1.02–7.23]) (Table 4).

In AD, we found an increased risk of viral infections in patients who received any JAKi (OR: 2.32 [1.46–3.69]) (Table 3). Once we performed our subgroup analyses, we found that selective JAKis were associated with increased risk of viral infections (OR: 2.60 [1.26–5.36]) as well as IL-4R targeted biologics (OR: 1.97 [1.07–3.61]) (Table 4).

In AA, we did not observe any difference in the risk of infection for both JAKi and biologics. In HS, there was no observed difference for biologic; however, there were no trials for JAKi in this patient population. There were no trials for either drug class in vitiligo patients that met our inclusion criteria.

### 3.5. Herpes Simplex and Herpes Zoster Infections

**Figure 5:**
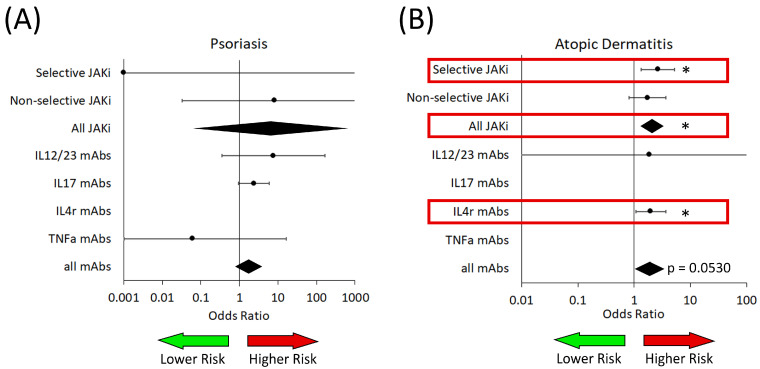
Risk of Herpes Simplex infection in patients from analyzed trials of both JAKi and biologic medications (mAbs). (**A**) Patients with psoriasis. (**B**) Patients with atopic dermatitis. IL—interleukin; TNFa—tumor necrosis factor alpha. The green arrow represents the area on the forest plot of reduced risk, while the red arrow represents the area of increased risk. Red boxes highlight significant associations with increased risk. Black diamonds are ORs for all JAK inhibitors and all biologics. * *p* < 0.05.

We examined the risk of herpes simplex and herpes zoster infections (Figure 5 and Table 5). There were no observed differences in the rates of herpes zoster infections between any of the drug classes in the main analysis and drug targets in the subgroup analysis, and placebo for both PsO and AD.

When we examine herpes simplex, we do not find any significant differences in the risk of infection in PsO patients. We do observe significant differences in the risk of herpes simplex infection in AD. Specifically, we found that receiving any type of JAKi was associated with an increased risk (OR: 2.08 [1.30–3.33]), with selective JAKi (OR: 2.66 [1.35–5.24]) and IL4R-targeted biologics having an increased risk (OR: 1.98 [1.07–3.65]) from our subgroup analysis.

## 4. Discussion

In this meta-analysis, we have shown that there is a significant infection risk associated with JAKi and biologics, which not only varies by type of pathogen (fungal, viral, and bacterial) but also by the skin condition (psoriasis, atopic dermatitis, vitiligo, hidradenitis suppurativa). To the best of our knowledge, this is the first meta-analysis to perform a detailed evaluation of clinical trial data to assess this risk across different disorders, including PsO, AD, vitiligo and HS, and different drugs (biologics and JAKi).

We have shown that fungal infections are more prevalent in PsO patients treated with IL-17 targeted biologics (Figure 2), which is consistent with previous reports [11,12,13,14]. IL-17 and Th17 cells are directly involved in immunity to fungi, with the inhibition of this pathway through -targeted biologics (brodalumab, bimekizumab, ixekizumab and secukinumab) allowing for these infections to take hold [15,16]. However, we did not see this association in patients with AD, alopecia areata or hidradenitis suppurativa, indicating that there may be disorder-specific characteristics that may affect risk in PsO patients. There were no trials that examined fungal infection risk in vitiligo. Fungal infections are known to be associated with PsO, and immune inhibition with-targeted biologics may provide an avenue for these infections to become apparent [17].

Bacterial infections (Figure 3 and Table 3) were found to be decreased in AD patients who had received dupilumab (IL4R-targeted biologic). This is a new observation for adult populations, but has been observed in pediatrics [18]. Dupilumab (IL-4R antagonist) inhibits allergic inflammation that occurs in AD, which can in turn promote responses that are favorable to bacterial clearance [19].

More specifically, IL-4R signaling through IL-4 and IL-13 is known to suppress both Th1 and Th17 immune responses [20,21,22,23,24]. Suppression of Th1 responses, particularly of classically activated M1 macrophages, with promotion of alternatively activated M2 macrophages through the activation of signal transducer and activator of transcription 6 (STAT6) leads to impaired bactericidal activity [21,22]. Inhibition of IL-4 signaling also promotes Th1 activation of CD8-positive T cells and improves their function, which aids in bacterial responses [24]. IL-4 impacts Th17 responses, inhibiting Th17 differentiation of T helper cells through the expression of GATA-3 binding protein, an inducer of the Th2 phenotype, which in turn downregulates the retinoic acid orphan receptor γt (RORγt), which is necessary for Th17 differentiation [21,25]. By inhibiting the Th2 response with drugs such as dupilumab, both the Th1 and Th17 responses can become more active, leading to more pro-inflammatory responses [25].

When we examined the rates of viral infections (Figure 4), we found an increased risk in AD patients exposed to JAKi. This was mainly in those receiving JAK1- and TYK2-selective drugs; however, we also observed a trend towards significance in those receiving non-selective JAKI (*p* = 0.0523), which may warrant further investigation in the future. We also found that PsO patients receiving any JAKi were at an increased risk of viral infection, which is consistent with work by Yiu et al. [26]. We observed that patients with PsO who received IL17-targeted biologics were also at increased risk of viral infection, which is a new observation (Figure 4 and Table 4).

Further investigations of viral infections for both herpes simplex and herpes zoster (Figure 5 and Table 5) demonstrated a significantly higher risk of herpes simplex infection in AD patients who received JAKi selective for JAK1 or TYK2 compared to placebo, a new observation, and for AD patients who received dupilumab (IL4R). The increased risk in AD patients is consistent with results from Ireland et al., who showed a relative increased risk of herpes simplex infection in AD patients compared to AA patients treated with JAKi [10]. JAK1 signaling is involved in many aspects of immune signaling, which can have significant but also varied effects on immunity through its impact on various members of the STAT family, namely STAT1, STAT3, STAT5 and STAT6 [27]. IL4 signaling is known to activate JAK1 signaling, which can impact antiviral responses [28,29]. Even though these new drugs are targeted to one specific molecule or pathway, many of these targets can have varied downstream effects on immune function, depending on the context of the disease in which they are activated [28,29,30]. IL4 signaling can activate type 2 responses, but also plays a role in stimulating the maturation of CD8+ and CD4+ T cells, particularly when the local environment is permissive [27,30].

When we take our results as a whole, we find a varied risk profile across drugs and disorders, which is consistent with previously published observations. Blauvelt et al. demonstrated that while there is no increased infection risk overall, they did find that herpetic infections were increased with dupilumab; however, no statistical tests were conducted to determine significance in their work [31]. Kridin et al. (2025), using the TriNetX dataset, examined herpetic and non-herpetic skin infection risk in AD patients prescribed dupilumab versus methotrexate and cyclosporine [32]. They found that non-herpetic skin infections, which would include bacterial infections, were decreased with dupilumab in a real-world population, which aligns with our work, but care should be taken in interpreting this work, as it is not placebo-controlled [32].

Fungal infections were found to be higher with bimekizumab (an IL17-targeted biologic) in work performed by Warren et al., but they also did not perform a statistical analysis to determine the significance of their work [33]. Ireland et al. examined the risk of varicella zoster infection, finding higher rates of infection with JAKi,, but there was no separation by disorder [10]. This particular result is contrary to our result; however, the inclusion and exclusion criteria for their study differ from ours in the inclusion of long-term extension data [10].

The work that we have presented provides valuable insight into the infection risks associated with these next-generation drugs. These drugs have been life-changing for many patients living with these dermatologic diseases; however, they are not without risk. Understanding these risks and how a particular patient may be impacted by each drug will enable health care providers to achieve optimal outcomes for their patients, as well as allow patients to make informed decisions regarding their care. Particular care should be taken when prescribing these medications, and patients should be screened for infections before and during treatment, with treatment stopped until any infections are resolved [34,35]. Additionally, these medications, being immunomodulatory, have the potential to impact vaccine efficacy through suppression of various immune pathways [36,37]. Live and live attenuated vaccines are not appropriate in patients prescribed these medications and are in fact not recommended in product monographs [34,35,37,38,39,40]. Miot et al. recommend that all vaccines should be updated prior to the initiation of JAKi [37].

That being said, there are limitations to this study that warrant discussion. Firstly, this meta-analysis has relied solely on clinical trial data. Including only trial data allowed us to have a comparison of infection risk within otherwise healthy individuals, apart from the disorder of interest. In the everyday clinical setting, patients will be of varied health status, including comorbid conditions and latent infections, which may give these patients a higher risk of infection and other complications from these drugs [12,13]. Studies such as those by Kridin et al. have used real-world data, such as that found within EHR records, which may more closely reflect the true infection rates [32,41]. Due to this limitation, the results and insights should be interpreted with care when considering these drugs for patients with compromised health, as infection risk may differ. An additional limitation is that the various trials may have used various definitions for each type of infection, which can influence results [10,26]. Also, some infections may be under-reported in some trials, which would lead to inaccuracies.

Another limitation related to the use of trial data is that the follow-up for these studies is relatively short, in some cases as short as 12 weeks (trials NCT02780167 and NCT03575871) (Ref the trials). This short length of time may not be long enough for some infections to fully manifest.

Finally, the paucity of trial data for some drugs in certain disorders also limits this present analysis. The low number of trials or lack of trials in AA, HS and vitiligo has the potential to limit the accuracy of those particular analyses.

## 5. Conclusions

The work presented highlights the varied infection risks that occur with the use of JAKi and biologic medications within different disorders. These factors should be considered when designing treatment plans for patients with PsO or AD. Additionally, the patient should be made aware of the specific infection risks for these drugs, enabling them to make informed decisions about their care. More research must be conducted to accurately determine these infection risks in HS, AA, and vitiligo in a similar manner to AD and PsO.

## Figures and Tables

**Table 1:** Number of patients (N) and Heterogeneity statistic (*I*^2^) for all comparisons.

	Psoriasis	Atopic Dermatitis	Alopecia Areata	Hidradenitis Suppurativa	Vitiligo
Fungal infection					
N	4147	3829	154	1957	n/a
*I* ^2^	22.5	34.2	0	29.4	n/a
Viral infection					
N	19746	8715	1348	1957	49
*I* ^2^	38.6	26.3	2.4	62.2	0
Bacterial infection					
N	18343	6630	60	1957	n/a
*I* ^2^	21.1	10.8	0	0.22	n/a
Simplex infection					
N	5359	8549	n/a	n/a	n/a
*I* ^2^	92.6	24.7	n/a	n/a	n/a
Zoster infection					
N	18402	4768	n/a	n/a	n/a
*I* ^2^	50.5	10.1	n/a	n/a	n/a

n/a—not applicable

**Table 2:** Summary of infection risk for JAKi and biologics in various dermatologic diseases.

	Psoriasis	Atopic Dermatitis	Alopecia Areata	Hidradenitis Suppurativa	Vitiligo
Fungal infections					
JAK inhibitors	OR: 4.88 (0.45–52.93) *p* = 0.2063	OR: 1.41 (0.28–7.19) *p* = 0.7217	OR: not estimable *p* = 0.9998	No trials	No trials
Biologics	**OR: 4.53 (2.34–8.78)** ***p* < 0.0001**	OR: 0.36 (0.03–6.30) *p* = 0.5158	OR: not estimable *p* = 0.9998	OR: not estimable *p* = 0.9967	No trials
Viral infections					
JAK inhibitors	**OR: 29.08** **(3.06–276.36)** ***p* = 0.0043**	**OR: 2.32 (1.46–3.69)** ***p* = 0.0013**	OR: 0.63 (0.27–1.43) *p* = 0.2036	No trials	Not enough data
Biologics	OR: 1.79 (0.84–3.79) *p* = 0.1129	**OR: 1.98 (1.12–3.49)** ***p* = 0.0395**	OR: not estimable *p* = 0.9851	OR: 2.35 (0.18–30.38) *p* = 0.4710	No trials
Bacterial infections					
JAK inhibitors	OR: 0.86 (0.11–7.03) *p* = 0.8899	OR: 1.21 (0.47–3.11) *p* = 0.8894	Not enough data	No trials	No trials
Biologics	OR: 1.29 (0.59–2.79) *p* = 0.5197	OR: 0.22 (0.06–0.88) *p* = 0.0534 *	Not enough data	OR: not estimable *p* = 0.9977	No trials

* Not significant after *p*-value adjustment for multiple comparisons. Bold cells represent significant findings.

**Table 3:** Summary of infection risk for different types of JAKi (selective and non-selective) in various dermatologic diseases.

	Psoriasis	Atopic Dermatitis	Alopecia Areata	Hidradenitis Suppurativa	Vitiligo
Fungal infections					
Selective for JAK1 or TYK2	OR: not estimable *p* = 0.1793	OR: not estimable *p* = 0.7304	OR: not estimable *p* = 0.9998	No trials	No trials
Not selective for specific JAK	OR: 3.544 (0.02–689.21) *p* = 0.9980	OR: 4.32 (0.27–70.36) *p* = 0.1236	No trials	No trials	No trials
Viral infections					
Selective for JAK1 or TYK2	OR: not estimable *p* = 0.3000	**OR: 2.6 (1.26–5.36)** ***p* = 0.0139**	OR: not estimable *p* = 0.8984	No trials	Not enough data
Not selective for specific JAK	OR: not estimable *p* = 0.0891	OR: 2.49 (1.04–5.98) *p* = 0.0523 *	OR: 0.59 (0.21–1.70) *p* = 0.2114	No trials	Not enough data
Bacterial infections					
Selective for JAK1 or TYK2	OR: not estimable *p* = 0.9971	OR: 0.74 (0.21–2.37) *p* = 0.5598	Not enough data	No trials	No trials
Not selective for specific JAK	OR: 0.88 (0.14–5.70) *p* = 0.8735	OR: 4.27 (0.52–35.33) *p* = 0.1291	Not enough data	No trials	No trials

* Not significant after *p*-value adjustment for multiple comparisons. Bold cells represent significant findings.

**Table 4:** Summary of infection risk with biologics to different targets in various dermatologic diseases.

	Psoriasis	Atopic Dermatitis	Alopecia Areata	Hidradenitis Suppurativa	Vitiligo
Fungal infections					
IL12/23 Targeted	OR: not estimable *p* = 0.9946	OR: not estimable *p* = 0.4689	No trials	No trials	No trials
IL17 Targeted	**OR: 6.07 (2.96–12.44)** ***p* < 0.0001**	No trials	No trials	OR: not estimable *p* = 0.9960	No trials
IL4R Targeted	No trials	OR: not estimable *p* = 0.9976	Not enough data	No trials	No trials
TNFa Targeted	OR: 0.52 (0.02–11.71) *p* = 0.6333	No trials	No trials	OR: not estimable *p* = 0.9617	No trials
Viral infections					
IL12/23 Targeted	OR: 6.70 (0.60–75.00) *p* = 0.1225	OR: not estimable *p* = 0.4963	No trials	No trials	No trials
IL17 Targeted	**OR: 2.72 (1.02–7.23)** ***p* = 0.0454**	No trials	No trials	OR: not estimable *p* = 0.9977	No trials
IL4R Targeted	No trials	OR: 1.97 (1.07–3.61) ***p* = 0.0312**	Not enough data	No trials	Not enough data
TNFa Targeted	OR: 0.06 (<0.001–17.15) *p* = 0.2786	No trials	No trials	OR: 4.35 (0.13–145.10) *p* = 0.3088	No trials
Bacterial infections					
IL12/23 Targeted	OR: 0.96 (0.33–2.80) *p* = 0.9294	OR: not estimable *p* = 0.9986	No trials	No trials	No trials
IL17 Targeted	OR 1.74 (0.65–4.67) *p* = 0.2668	No trials	No trials	Not enough data	No trials
IL4R Targeted	No trials	**OR: 0.23 (0.06–0.92)** ***p* = 0.0394**	Not enough data	No trials	No trials
TNFa Targeted	OR: 1.06 (0.25–4.52) *p* = 0.8190	No trials	No trials	OR: not estimable *p* = 1.0	No trials

Not significant after *p*-value adjustment for multiple comparisons. Bold cells represent significant findings.

**Table 5:** Risk of Herpes Simplex and Herpes Zoster Infection with JAKi and Biologics in Psoriasis and Atopic Dermatitis.

	Herpes Simplex		Herpes Zoster	
	Psoriasis	AD	Psoriasis	AD
JAK inhibitors				
Selective for JAK1 or TYK2	OR: not estimable *p* = 0.9982	**OR: 2.66 (1.35–5.24)** ***p* = 0.0084**	OR: not estimable *p* = 0.9978	OR: not estimable *p* = 0.9910
Not selective for specific JAK	OR: not estimable *p* = 0.3989	OR: 1.73 (0.81–3.69) *p* = 0.1321	OR: not estimable *p* = 0.9954	OR: not estimable *p* = 0.9979
All JAK inhibitors	OR: 6.60 (0.07–666.38) *p* = 0.3834	**OR: 2.08 (1.30–3.33)** ***p* = 0.0061**	OR: not estimable *p* = 0.9978	OR: not estimable *p* = 0.9978
Biologics				
IL12/23 targeted	OR: 7.68 (0.36–165.23) *p* = 0.1762	OR: not estimable *p* = 0.6804	OR: not estimable *p* = 0.9581	OR: not estimable *p* = 0.9987
IL17 targeted	OR: 2.38 (0.94–6.01) *p* = 0.0663	No trials	Not enough data ^†^	No trials
IL4r targeted	No trials	**OR: 1.98 (1.07–3.65)** ***p* = 0.0312**	No trials	OR: not estimable *p* = 0.9674
TNFa targeted	OR: not estimable *p* = 0.2760	No trials	OR: not estimable *p* = 0.9996	No trials
All biologics	OR: 1.74 (0.78–3.87) *p* = 0.1710	OR: 1.90 (1.08–3.34) *p* = 0.0530 *	OR: not estimable *p* = 0.9479	OR: not estimable *p* = 0.9981

* Not significant after *p*-value adjustment for multiple comparisons. ^†^ Accurate odds ratio could not be calculated. Bold cells represent significant findings.

## Data Availability

Original data for all trials included in this work can be found at clinicaltrials.gov.

## Supplementary Materials

The following supporting information can be downloaded at: https://www.mdpi.com/article/10.3390/medicina61112053/s1; Table S1: Systematic search queries; Table S2: Inclusion and exclusion criteria; Figure S1: Doi Plots for Assessing Publication Bias; Table S3: Trial characteristics with NCT number (clinicaltrials.gov); Table S4: Risk of bias assessment.

## Abbreviations

The following abbreviations are used in this manuscript:

AA	Alopecia areata
AD	Atopic dermatitis
HS	Hidradenitis suppurativa
PsO	Psoriasis
OR	Odds Ratio
JAKi	Janus Kinase inhibitors
TYK2	Tyrosine Kinase 2
IL	Interleukin
DMARDs	Disease Modifying Anti-Rheumatic Drugs
STAT	Signal Transducer and Activator of Transcription
CD	Cluster of Differentiation
RORγt	retinoic acid orphan receptor γt
GATA-3	G-A-T-A—3 binding protein

## References

[1] Curtis K.L., Stubblefield O., Lipner S.R. (2024). Alopecia Areata Is Associated with Posttraumatic Stress Disorder and Alcohol Use in a Case-Control Study of 4785 Patients. Skin. Appendage Disord..

[2] Feldman S.R., Gomez B., Meng X., Germino R. (2021). Secukinumab rapidly improves EQ-5D health status in patients with psoriasis: Pooled analysis from four phase 3 trials. J. Dermatol. Treat..

[3] Tsai Y.C., Hung C.Y., Tsai T.F. (2023). Efficacy and Safety of Biologics and Small Molecules for Moderate-to-Severe Hidradenitis Suppurativa: A Systematic Review and Network Meta-Analysis. Pharmaceutics.

[4] Clemmesen M.E.R., Gren S.T., Frøstrup A.G., Thomsen S.F., Egeberg A., Thein D. (2024). Psychosocial and mental impact of alopecia areata: Analysis of the Danish Skin Cohort. J. Eur. Acad. Dermatol. Venereol..

[5] Reed B., Blaiss M.S. (2018). The burden of atopic dermatitis. Allergy Asthma Proc..

[6] Benton S., Farah R., Hordinsky M. (2023). Systemic Immunotherapies.

[7] Kim E.S., Garnock-Jones K.P., Keam S.J. (2016). Adalimumab: A Review in Hidradenitis Suppurativa. Am. J. Clin. Dermatol..

[8] Ibrahim A., Ahmed M., Conway R., Carey J.J. (2019). Risk of infection with methotrexate therapy in inflammatory diseases: A systematic review and meta-analysis. J. Clin. Med..

[9] Wilson D.L., Zhou L., Sudano D.G., Ashbeck E.L., Kwoh C.K., Krebs L., Sheer A., Smith J., Tudeen M., Lo-Ciganic W.H. (2024). Risk of Coccidioidomycosis Infection Among Individuals Using Biologic Response Modifiers, Corticosteroids, and Oral Small Molecules. ACR Open Rheumatol..

[10] Ireland P.A., Verheyden M., Jansson N., Sebaratnam D., Sullivan J. (2025). Infection risk with JAK inhibitors in dermatoses: A meta-analysis. Int. J. Dermatol..

[11] Yamanaka-Takaichi M., Ghanian S., Katzka D.A., Torgerson R.R., Alavi A. (2022). Candida Infection Associated with Anti-IL-17 Medication: A Systematic Analysis and Review of the Literature. Am. J. Clin. Dermatol..

[12] Bahr N.C., Benedict K., Toda M., Gold J.A.W., Lipner S.R. (2024). Low incidence of invasive fungal infections in a large observational cohort of patients initiating IL-17 or IL-23 inhibitor therapy, United States, 2016–2022. J. Am. Acad. Dermatol..

[13] Schwarz C.W., Näslund-Koch C., Zachariae C., Seidelin J.B., Nielsen S.D., Ostrowski S.R., Astvad K.M.T., Brock I., Iversen L., Rasmussen M.K. (2025). Adverse Events and Immune Response in Psoriasis Patients Receiving Interleukin-17 Inhibitors. Acta Derm. Venereol..

[14] Minami Y., Hiruma J., Harada K., Fujimori K., Suzuki R., Mori M., Okura M., Abe N., Harada K., Okubo Y. (2025). Risk of fungal infection in patients with psoriasis receiving biologics: A retrospective single-center cohort study. J. Am. Acad. Dermatol..

[15] Bi Y., Liu G., Yang R. (2011). Reciprocal modulation between TH17 and other helper T cell lineages. J. Cell Physiol..

[16] Ruchti F., LeibundGut-Landmann S. (2023). New insights into immunity to skin fungi shape our understanding of health and disease. Parasite Immunol..

[17] Zhou S., Yao Z. (2022). Roles of Infection in Psoriasis. Int. J. Mol. Sci..

[18] Paller A.S., Siegfried E.C., Cork M.J., Arkwright P.D., Eichenfield L.F., Ramien M., Khokhar F.A., Chen Z., Zhang A., Cyr S.L. (2024). Infections in Children Aged 6 Months to 5 Years Treated with Dupilumab in a Placebo-Controlled Clinical Trial of Moderate-to-Severe Atopic Dermatitis. Pediatr. Drugs.

[19] Leyva-Castillo J.M., McGurk A., Strakosha M., Vega-Mendoza D., Smith S.E.M., Stafstrom K., Elkins M., Chou J., Wang Y.H., Geha R.S. (2023). IL-4 receptor alpha blockade dampens allergic inflammation and upregulates IL-17A expression to promote S aureus clearance in antigen sensitized mouse skin. J. Allergy Clin. Immunol..

[20] Guenova E., Skabytska Y., Hoetzenecker W., Weindl G., Sauer K., Tham M., Kim K.W., Park J.H., Seo J.H., Ignatova D. (2015). IL-4 abrogates TH17 cell-mediated inflammation by selective silencing of IL-23 in antigen-presenting cells. Proc. Natl. Acad. Sci. USA.

[21] Luzina I.G., Keegan A.D., Heller N.M., Rook G.A.W., Shea-Donohue T., Atamas S.P. (2012). Regulation of inflammation by interleukin-4: A review of “alternatives”. J. Leukoc. Biol..

[22] Bao K., Reinhardt R.L. (2015). The differential expression of IL-4 and IL-13 and its impact on type-2 immunity. Cytokine.

[23] Lundahl M.L.E., Mitermite M., Ryan D.G., Case S., Williams N.C., Yang M., Lynch R.I., Lagan E., Lebre F.M., Gorman A.L. (2022). Macrophage innate training induced by IL-4 and IL-13 activation enhances OXPHOS driven anti-mycobacterial responses. eLife.

[24] Ranasinghe C., Trivedi S., Wijesundara D.K., Jackson R.J. (2014). IL-4 and IL-13 receptors: Roles in immunity and powerful vaccine adjuvants. Cytokine Growth Factor. Rev..

[25] Bridgewood C., Newton D., Bragazzi N., Wittmann M., McGonagle D. (2021). Unexpected connections of the IL-23/IL-17 and IL-4/IL-13 cytokine axes in inflammatory arthritis and enthesitis. Semin. Immunol..

[26] Yiu Z.Z.N., Exton L.S., Jabbar-Lopez Z., Mohd Mustapa M.F., Samarasekera E.J., Burden A.D., Murphy R., Owen C.M., Parslew R., Venning V. (2016). Risk of Serious Infections in Patients with Psoriasis on Biologic Therapies: A Systematic Review and Meta-Analysis. J. Investig. Dermatol..

[27] Hu Q., Bian Q., Rong D., Wang L., Song J., Huang H.S., Zeng J., Mei J., Wang P.Y. (2023). JAK/STAT pathway: Extracellular signals, diseases, immunity, and therapeutic regimens. Front. Bioeng. Biotechnol..

[28] Howard F.H.N., Kwan A., Winder N., Mughal A., Collado-Rojas C., Muthana M. (2022). Understanding Immune Responses to Viruses—Do Underlying Th1/Th2 Cell Biases Predict Outcome?. Viruses.

[29] Brooks W.H., Renaudineau Y. (2024). Not just another klass (JAK) of inhibitors for allergies. J. Allergy Hypersensit. Dis..

[30] Xue C., Yao Q., Gu X., Shi Q., Yuan X., Chu Q., Bao Z., Lu J., Li L. (2023). Evolving cognition of the JAK-STAT signaling pathway: Autoimmune disorders and cancer. Signal Transduct. Target. Ther..

[31] Blauvelt A., Wollenberg A., Eichenfield L.F., Zhang H., Sierka D., Khokhar F.A., Vakil J., Shabbir A., Marco A.R., Cyr S.L. (2023). No Increased Risk of Overall Infection in Adults with Moderate-to-Severe Atopic Dermatitis Treated for up to 4 Years with Dupilumab. Adv. Ther..

[32] Kridin K., Abdelghaffar M., Bieber K., Thaci D., Ludwig R.J. (2025). The real-world, long-term risk of infections associated with dupilumab in atopic dermatitis: A global cohort study. J. Eur. Acad. Dermatol. Venereol..

[33] Warren R.B., Lebwohl M., Thaçi D., Gooderham M., Pinter A., Paul C., Gisondi P., Szilagyi B., White K., Deherder D. (2025). Bimekizumab efficacy and safety through 3 years in patients with moderate-to-severe plaque psoriasis: Long-term results from the BE RADIANT phase IIIb trial open-label extension period. Br. J. Dermatol..

[34] Sanofi-Aventis US LLC DUPIXENT^®^ (Dupilumab) Injection, for Subcutaneous Use. 2025. https://www.accessdata.fda.gov/drugsatfda_docs/label/2025/761055s070lbl.pdf.

[35] AbbVie SKYRIZI^®^ (Risankizumab-Rzaa) Injection, for Subcutaneous or Intravenous Use. 2024. https://www.accessdata.fda.gov/drugsatfda_docs/label/2025/761105s039,761262s011lbl.pdf.

[36] Ergun T., Hosgoren Tekin S., Apti Sengun O., Akin Cakici O., Seckin D., Adiay C., Enul H., Yilmaz S., Ay P., Haklar G. (2023). Immunogenicity, efficacy, and safety of CoronaVac and Pfizer/BioNTech mRNA vaccines in patients with psoriasis receiving systemic therapies: A prospective cohort study. Vaccine.

[37] Miot H.A., Criado P.R., de Castro C.C.S., Ianhez M., Talhari C., Ramos P.M. (2023). JAK-STAT pathway inhibitors in dermatology. An. Bras. Dermatol..

[38] Pfizer Laboratories Div Pfizer Inc XELJANZ (Tofacitinib) Tablets, for Oral Use; XELJANZ XR (Tofacitinib) Extended-Release Tablets, for Oral Use; XELJANZ (Tofacitinib) Oral Solution. 2025. https://www.accessdata.fda.gov/drugsatfda_docs/label/2020/203214s026lbl.pdf.

[39] Pfizer Labs LITFULO^TM^ (Ritlecitinib) Capsules, for Oral Use. 2023. https://www.accessdata.fda.gov/drugsatfda_docs/label/2023/215830s000lbl.pdf.

[40] FDA, CDER TREMFYA^®^ (Guselkumab) Injection, for Subcutaneous or Intravenous Use. https://www.accessdata.fda.gov/drugsatfda_docs/label/2025/761061s026lbl.pdf.

[41] Kridin K., Zirpel H., Mruwat N., Ludwig R.J., Thaci D. (2023). Evaluating the risk of infections under interleukin 23 and interleukin 17 inhibitors relative to tumour necrosis factor inhibitors—A population-based study. J. Eur. Acad. Dermatol. Venereol..

