# Supplementary material for: Infection Risk in Dermatology Patients Receiving Next-Generation Medication: A Meta-Analysis of JAK Inhibitors and Biologics
Source: Medicina (Kaunas). 2025 Nov 18;61(11):2053. doi: 10.3390/medicina61112053 (PMC12654054; doi:10.3390/medicina61112053)
Supplement: Supplementary file 1 [file medicina-61-02053-s001.zip › medicina-3931103-supplementary.pdf]

Table S1 – Systematic search queries

Database	Query String
PubMed	
	(‘small molecule inhibitors’ OR 'JAK inhibitors') AND ('Alopecia areata' OR 'acne inversa' OR 'Hidradenitis suppurativa' OR Psoriasis OR 'Atopic dermatitis' OR Vitiligo) – filter for: ‘Randomized Controlled Trial’ and English
	‘Biologics’ AND ('Alopecia areata' OR 'acne inversa' OR 'Hidradenitis suppurativa' OR Psoriasis OR 'Atopic dermatitis' OR Vitiligo) – filter for: ‘Randomized Controlled Trial’ and English
Web of Science	
	(TS=("small molecule inhibitors" OR "JAK inhibitors")) AND (TS=("Alopecia areata" OR "acne inversa" OR "Hidradenitis suppurativa" OR Psoriasis OR "Atopic dermatitis" OR Vitiligo)) AND (DT="Randomized Controlled Trial")
	(TS=Biologics) AND (TS=("Alopecia areata" OR "acne inversa" OR "Hidradenitis suppurativa" OR Psoriasis OR "Atopic dermatitis" OR Vitiligo)) AND (DT="Randomized Controlled Trial")
Scopus	
	(TITLE-ABS-KEY("small molecule inhibitors" OR "JAK inhibitors")) AND (TITLE-ABS-KEY("Alopecia areata" OR "acne inversa" OR "Hidradenitis suppurativa" OR Psoriasis OR "Atopic dermatitis" OR Vitiligo))
	(TITLE-ABS-KEY(Biologics)) AND (TITLE-ABS-KEY("Alopecia areata" OR "acne inversa" OR "Hidradenitis suppurativa" OR Psoriasis OR "Atopic dermatitis" OR Vitiligo))
Clinicaltrials.gov	
	Condition/Disease (Alopecia areata, acne inversa, Hidradenitis suppurativa, Psoriasis, Atopic dermatitis, Vitiligo)<br>Intervention/treatment (JAK inhibitor treatment, biologic treatment)<br><br>Filter for: completed trials, trials with results

Table S2 – Inclusion and exclusion criteria

Include	Exclude
• Studies of patients with the following (as the primary condition):<br>○ Atopic dermatitis<br>○ Psoriasis<br>○ Alopecia areata<br>○ Hidradenitis suppurativa<br>○ Vitiligo<br>• Randomized clinical trials – Phase 2 and Phase 3<br>• Multi-center studies<br>• Interventions:<br>○ Biologics<br>○ JAK inhibitors<br>• Concurrent topical therapies<br>• Placebo-controlled trials<br>• Blinded trials<br>• Outcomes:<br>○ Fungal infections—dermatophyte and candida<br>○ Viral infections—herpes simples and herpes zoster, general herpes infection<br>○ Bacterial infections—staphylococcus aureus infection, cellulitis, other bacterial infections	• Studies of patients with the following (as the primary condition):<br>○ Arthritic disorders<br>○ Ulcerative colitis<br>○ Crohn's disease<br>○ Other non-dermatological conditions<br>• Meta-analyses<br>• Reviews<br>• Post-marketing trials<br>• Trials with no placebo comparator<br>• Biosimilars<br>• Open-label trials<br>• Cross-over studies (latter arms)<br>• Concurrent oral therapies<br>○ Oral corticosteroids and other DMARDs<br>• Studies that do not list our outcomes of interest

Figure S1 – Doi Plots for Assessing Publication Bias. (A) Fungal Infections. (B) Bacterial Infections. (C) Viral infections.

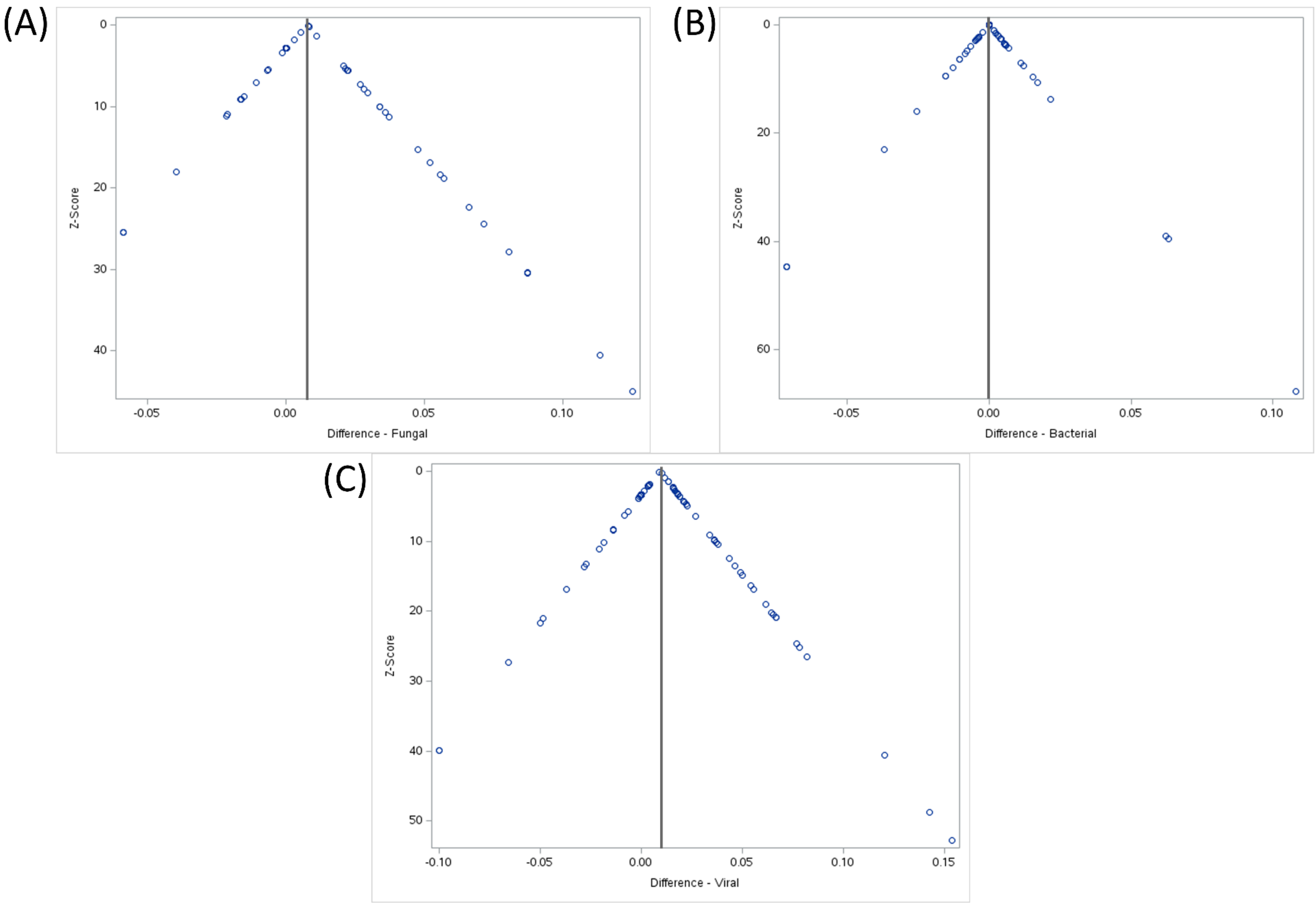

Table S3 – Trial characteristics with NCT number (clinicaltrials.gov)

Trial Name/ NCT number	Trial Phase	Disorder	Blinding	Allocation method	Drug	Dosing Regime	Route of Administration	Additional concurrent treatments	Patients in trial arm	Age, mean (SD)	Females (n)	Males (n)	Treatment Duration (weeks)	AE evaluation timepoint
NCT02201524	Phase 2	Psoriasis	double-blind	randomized	placebo	0 mg once daily	oral		14	45.4 (10.8)	3	11	4 weeks	Not specified
					abrocitinib	200 mg once daily	oral		15	42.9(12.3)	5	10	4 weeks	Not specified
					abrocitinib	400 mg once daily	oral		16	42.8(11.6)	5	11	4 weeks	Not specified
					abrocitinib	200 mg twice daily	oral		14	52.1(9.7)	6	8	4 weeks	Not specified
NCT02576938	Phase 2	AD	double-blind	randomized	placebo	0 mg	oral	Topical Corticosteroids	49	37.3(13.49)	25	24	16 weeks	up to 20 weeks
					baricitinib	2 mg	oral	Topical Corticosteroids	37	40.0(14.38)	15	22	16 weeks	up to 20 weeks
					baricitinib	4 mg	oral	Topical Corticosteroids	38	36.4(14.47)	16	22	16 weeks	up to 20 weeks
NCT02780167	Phase 2B	AD	double-blind	randomized	placebo	0 mg	oral		56	42.6(15.1)	35	21	12 weeks	12 weeks
					abrocitinib	10 mg	oral		49	44.3(15.9)	28	21	12 weeks	12 weeks

					abrocitinib	30 mg	oral		51	37.6(15.9)	29	22	12 weeks	12 weeks
					abrocitinib	100 mg	oral		56	41.1(15.6)	35	31	12 weeks	12 weeks
					abrocitinib	200 mg	oral		55	38.7(17.6)	27	28	12 weeks	12 weeks
NCT02925117	Phase 2B	AD	double-blind	randomized	placebo	0mg	oral		41	39.9(17.52)	17	24	16 weeks	16 weeks
					Upadacitinib	7.5 mg	oral		42	41.5(15.36)	14	28	16 weeks	16 weeks
					Upadacitinib	15 mg	oral		42	38.5(15.24)	12	30	16 weeks	16 weeks
					Upadacitinib	30 mg	oral		42	39.9(15.30)	20	22	16 weeks	16 weeks
BREEZE-AD2/NCT03334422	Phase 3	AD	double-blind	randomized	placebo	0 mg	oral		244	<=18: 0<br>18-65: 235<br>>=65: 9	90	154	16 weeks	up to 20 weeks
					baricitinib	1 mg	oral		125	<=18: 0<br>18-65: 124<br>>=65: 1	45	80	16 weeks	up to 20 weeks
					baricitinib	2 mg	oral		123	<=18: 0<br>18-65: 118<br>>=65: 5	58	65	16 weeks	up to 20 weeks
					baricitinib	4 mg	oral		123	<=18: 0<br>18-65: 119<br>>=65: 4	41	82	16 weeks	up to 20 weeks
BREEZE-AD4/NCT03428100	Phase 3	AD	double-blind	randomized	placebo	0 mg	oral	Topical Corticosteroids	93	38.7(13.6)	44	49	16 weeks	up to 200 weeks
					baricitinib	1 mg	oral	Topical Corticosteroids	93	38.9(14.0)	35	58	16 weeks	up to 200 weeks

					baricitinib	2 mg	oral	Topical Corticosteroids	185	37.3(13.6)	52	133	16 weeks	up to 200 weeks
					baricitinib	4 mg	oral	Topical Corticosteroids	92	38.7(13.3)	35	57	16 weeks	up to 200 weeks
AD Up/ NCT03568318	Phase 3	AD	double-blind	randomized	Placebo	0mg	oral	Topical corticosteroids	264	37.2(14.08)	101	163	16 weeks	16 weeks
					Upadacitinib	15 mg	oral	Topical corticosteroids	261	35(13.29)	104	157	16 weeks	16 weeks
					Upadacitinib	30 mg	oral	Topical corticosteroids	260	28.3(14.82)	95	165	16 weeks	16 weeks
Measure up 1/ NCT03569293	Phase 3	AD	double-blind	randomized	placebo	0mg	oral		241	37.6(14.44)	114	127	16 weeks	16 weeks
					Upadacitinib	15mg	oral		239	37.3(14.80)	103	136	16 weeks	16 weeks
					Upadacitinib	30mg	oral		243	36.7(15.12)	110	133	16 weeks	16 weeks
BRAVE-AA1/NCT03570749	Phase 3	AA	double-blind	randomized	placebo	0 mg	oral		189	37.4(12.9)	109	80	36 weeks	36 weeks
					baricitinib	2 mg	oral		184	38(12.8)	109	75	36 weeks	36 weeks
					baricitinib	4 mg	oral		281	36.3(13.3)	165	116	36 weeks	36 weeks

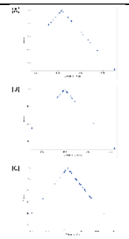

JADE MONO/NCT03575871	Phase 3	AD	double blind	randomized	abrocitinib	100 mg	oral	158	37.4(15.8)	64	94	12 weeks	16 weeks
					abrocitinib	200 mg	oral	155	33.5(14.7)	67	88	12 weeks	16 weeks
					placebo	placebo	oral	78	33.4(13.8)	31	47	12 weeks	16 weeks
Measure up 2/ NCT03607422	Phase 3	AD	double-blind	randomized	placebo	0mg	oral	242	36.0(14.08)	104	138	16 weeks	16 weeks
					Upadacitinib	15mg	oral	243	35.7(15.15)	102	141	16 weeks	16 weeks
					Upadacitinib	30mg	oral	247	36.7(15.36)	103	144	16 weeks	16 weeks
JADE COMPARE/NCT03720470	Phase 3	AD	double blind	randomized	placebo	0 mg	injection	131	<=18: 0<br>18-65: 121<br>>=65: 0	54	77	16 weeks	16 weeks
					abrocitinib	100 mg	oral	238	<=18: 0<br>18-65: 224<br>>=65: 14	118	120	16 weeks	16 weeks
					abrocitinib	200 mg	oral	226	<=18: 0<br>18-65: 211<br>>=65: 15	122	104	16 weeks	16 weeks
					dupilumab	300 mg	SC	243	<=18: 0<br>18-65: 211<br>>=65: 15	122	104	16 weeks	16 weeks
Heads up/ NCT03738397	Phase 3	AD	double-blind	randomized	dupilumab	300mg	SC	331	36.3(13.81)	139	192	16 weeks	**16 weeks**
					Upadacitinib	30mg	oral	342	36.2(14.42)	159	183	16 weeks	**16 weeks**

Jade TEEN/NCT03796676	Phase 3	AD	double-blind	randomized	placebo	0 mg	oral	96	14.8(1.7)	52	44	12 weeks	16 weeks
					abrocitinib	100 mg	oral	95	15.1(1.8)	50	45	12 weeks	16 weeks
					abrocitinib	200 mg	oral	94	14.7(1.8)	38	56	12 weeks	16 weeks
BRAVE-AA2/ NCT03899259	Phase 3	AA	double-blind	randomized	placebo	0mg	oral	156	37.1(12.4)	98	58	36 weeks	36 weeks
					Baricitinib	2mg	oral	156	39.0(13.0)	103	53	36 weeks	36 weeks
					Baricitinib	4mg	oral	234	28.0(12.7)	144	90	36 weeks	36 weeks
Jade MOA/NCT03915496	Phase 2a	AD	double-blind	randomized	placebo	0 mg	oral	16	38.9(15.64)	3	13	12 weeks	16 weeks
Jade MOA/NCT03915496	Phase 2a	AD	double-blind	randomized	abrocitinib	100 mg	oral	16	46.6(19.41)	11	5	12 weeks	16 weeks
					abrocitinib	200 mg	oral	14	41.4(16.72)	7	7	12 weeks	16 weeks
BREEZE-AD-PEDS/NCT03952559	Phase 3	AD	double-blind	randomized	placebo	0 mg	oral	122	11.75(4.012)	64	58	16 weeks	16 weeks
					baricitinib	low dose: 10 - < 18 years: 1 mg 2 - < 10 years: 0.5 mg	oral	121	12.35(4.052)	62	59	16 weeks	16 weeks
					baricitinib	medium dose: 10 - < 18 years: 2 mg 2 - < 10 years: 1 mg	oral	120	11.81(3.661)	63	57	16 weeks	16 weeks

					baricitinib	high dose: 10 - < 18 years: 4 mg 2 - < 10 years: 2 mg	oral		120	11.93(3.829)	53	67	16 weeks	16 weeks
Jade DARE/NCT04345367	Phase 3b	AD	double-blind	randomized	abrocitinib	200 mg	oral		362	36.6(14.6)	169	193	26 weeks	30 weeks
						300 mg	SC		365	35.5(13.3)	161	204	24 weeks	30 weeks
NCT04822584	Phase 2	vitiligo	double-blind	randomized	placebo	0 mg	oral	narrow band UV-B phototherapy	12	median: 47.1 (36.9-52.6)	7	5	36 weeks	up to 36 weeks
					baricitinib	4 mg	oral	narrow band UV-B phototherapy	37	median: 52.3 (42-60.8)	28	9	36 weeks	up to 36 weeks
					baricitinib	4 mg	oral	narrow band UV-B phototherapy	37	median: 52.3 (42-60.8)	28	9	36 weeks	up to 36 weeks
					baricitinib	4 mg	oral	narrow band UV-B phototherapy	37	median: 52.3 (42-60.8)	28	9	36 weeks	up to 36 weeks
NCT05556265	Phase 2	AA	double-blind	randomized	placebo	0mg	oral		31	28.9(14.40)	20	11	24 weeks	24 weeks
					Deucravacitinib	6mg QD	oral		32	36.1(13.37)	22	10	24 weeks	24 weeks
					Deucravacitinib	6mg BID	oral		31	43.4(13.81)	22	9	24 weeks	24 weeks

PEDS/ NCT03568318	Phase 3	AD	double-blind	randomized	placebo	0 mg	oral	Topical corticosteroids	63	15.1(1.85)	36	27	16 weeks	16 weeks
					Upadacitinib	15 mg	oral	Topical corticosteroids	60	15.4(1.65)	27	33	16 weeks	16 weeks
					Upadacitinib	30 mg	oral	Topical corticosteroids	60	15.3(1.86)	25	35	16 weeks	16 weeks
Measure up 1/ NCT03569293	Phase 3	AD	double-blind	randomized	placebo	0mg	oral		61	15.1(1.70)	33	28	16 weeks	16 weeks
					Upadacitinib	15mg	oral		64	15.5(1.99)	34	30	16 weeks	16 weeks
					Upadacitinib	30mg	oral		64	15.7(1.63)	36	28	16 weeks	16 weeks
Measure up 2/ NCT03607422	Phase 3	AD	double-blind	randomized	placebo	0mg	oral		60	15.5(1.67)	35	25	16 weeks	16 weeks
					Upadacitinib	15mg	oral		58	15.2(1.79)	38	20	16 weeks	16 weeks
					Upadacitinib	30mg	oral		62	15.8(1.70)	26	36	16 weeks	16 weeks
PIONEER I/NCT01468207	3	HS	double	randomized	Placebo		SC	-	154	37.8 (11.33)	105	49	12 weeks	12 weeks
					adalimumab	160 mg at Week 0; 80 mg at Week 2; and 40 mg ew from Week 4 to Week 12	SC	-	153	36.2 (10.83)	91	62	12 weeks	12 weeks
PIONEER II/NCT01468233	3	HS	double	randomized	Placebo		SC	-	163	36.1 (12.18)	113	50	12 weeks	12 weeks

					adalimumab	160 mg at Week 0; 80 mg at Week 2; and 40 mg ew from Week 4 to Week 12	SC	-	163	34.9 (9.96)	108	55	12 weeks	12 weeks
NCT00918255	2	HS	double	randomized	adalimumab	160 mg at Week 0, adalimumab 80 mg at Week 2, 40 mg Qwk DB	SC	-	51	35.1 (10.69)	36	15	16 weeks	16 weeks
					adalimumab	adalimumab 80 mg at Week 0, followed by adalimumab 40 mg eow	SC	-	53	36.1 (12.50)	38	14	16 weeks	16 weeks
					placebo		SC	-	51	37.8 (12.10)	36	15	16 weeks	16 weeks
NCT01251614	3	PSO	double	randomized	Methotrexate	0.1 mg/kg	Oral	-	37	13.4 (3.49)	26	11	16 weeks	16 weeks
					adalimumab	0.4mg/kg	SC	-	39	12.6 (4.43)	18	21	16 weeks	16 weeks
					adalimumab	0.8mg/kg	SC	-	22	13.0 (3.29)	21	17	16 weeks	16 weeks
NCT03248531	2	HS	double	randomized	Placebo		SC	-	22	40.7 (12.5)	15	7	12 weeks	12 weeks
					adalimumab	160 mg	SC	-	22	31.0 (9.2)	18	4	12 weeks	12 weeks
					Bimekizumab	640 mg	SC	-	46	37.4 (11.9)	30	16	12 weeks	12 weeks

VOYAGE<br>1/NCT02207231	3	PSO	double	randomized	Placebo		SC	-	174	44.9<br>(12.9)	55	119	16 weeks	16 weeks
					Guselkum<br>ab	100mg	SC	-	329	43.9<br>(12.74)	89	240	16 weeks	16 weeks
					Adalimum<br>ab	40 mg	SC	-	334	42.9<br>(12.58)	85	249	16 weeks	16 weeks
VOYAGE<br>2/NCT02207244	3	PSO	double	randomized	Placebo		SC	-	248	43.3<br>(12.38)	75	173	16 weeks	16 weeks
				randomized	Guselkum<br>ab	100mg	SC	-	496	43.7<br>(12.23)	14<br>7	349	16 weeks	16 weeks
				randomized	Adalimum<br>ab	80mg	SC	-	248	43.2<br>(11.92)	78	170	16 weeks	16 weeks
NCT02694523	3	PSO	double	randomized	adalimum<br>ab	80mg	SC	-	302	47.0<br>(13.09)	92	212	16 weeks	16 weeks
					risankizu<br>mab	150mg	SC	-	301	45.3<br>(13.79)	91	210	16 weeks	16 weeks
JADE<br>COMPARE/NCT0<br>3720470	3	AD	double	randomized	Placebo		SC	-	131	<=18: 0<br>18-65:<br>121<br>>=65: 0	54	77	16 weeks	16 weeks
					Abrocitinib	100mg	oral	-	238	<=18: 0<br>18-65:<br>224<br>>=65: 14	11<br>8	120	16 weeks	16 weeks
					Abrocitinib	200mg	oral	-	226	<=18: 0<br>18-65:<br>211<br>>=65: 15	12<br>2	104	16 weeks	16 weeks
					Dupiluma<br>b	300mg	SC	-	243	<=18: 0<br>18-65:<br>211<br>>=65: 15	13<br>4	108	16 weeks	16 weeks
Heads up/<br>NCT03738397	3b	AD	double	randomized	Dupiluma<br>b	300mg	SC	-	331	36.3<br>(13.81)	13<br>9	192	24 weeks	24 weeks
					Upadaciti<br>nib	30mg	oral	-	342	36.2<br>(14.42)	15<br>9	183	24 weeks	24 weeks

Liberty AD PRESCHOOL/NCT03346434	3	AD	double	randomized	Placebo		SC	TCS	79	6 months - <2 years: 5 ≥2 years and <6 years: 74	24	55	16 weeks	16 weeks
					Dupilumab	200/300mg	SC	TCS	83	6 months - <2 years: 6 ≥2 years and <6 years: 747	39	44	16 weeks	16 weeks
NCT04678882	3	AD	double	randomized	Placebo		SC	TCS	32	9.6 ± 4.2	11	21	16 weeks	16 weeks
					Dupilumab	≥5 to <15 kg: 200mg ≥15 to <30 kg: 300mg	SC	TCS	30	10.0 ± 4.1	12	18	16 weeks	16 weeks
NCT04345367	3b	AD	double	randomized	Abrocitinib	200mg	oral	TCS	362	36.6 (14.6)	169	193	16 weeks	16 weeks
					Dupilumab	300mg	SC	TCS	365	35.5 (13.3)	161	204	16 weeks	16 weeks
SOLO-1/NCT02277743	3	AD	double	randomized	Placebo		SC	-	224	39.5 (13.91)	106	118	16 weeks	16 weeks
					Dupilumab	300mg q2w	SC	-	224	39.8 (14.68)	94	130	16 weeks	16 weeks
					Dupilumab	300mg qw	SC	-	223	39.3 (14.39)	81	142	16 weeks	16 weeks
SOLO 2/NCT02277769	3	AD	double	randomized	Placebo		SC	-	236	37.4 (14.09)	104	132	16 weeks	16 weeks
					Dupilumab	300mg q2w	SC	-	233	36.9 (13.96)	96	137	16 weeks	16 weeks

					Dupilumab	300mg qw	SC	-	239	37.1 (14.51)	100	139	16 weeks	16 weeks
SOLO-CONTINUE/NCT02395133	3	AD	double	randomized	Placebo		SC	-	83	38.1 (13.64)	32	51	36 weeks	36 weeks
					Dupilumab	300 mg q8w	SC	-	84	37.3 (13.98)	33	51	36 weeks	36 weeks
					Dupilumab	300 mg q4w	SC	-	86	38.5 (16.76)	43	43	36 weeks	36 weeks
					Dupilumab	300mg q2w/qw	SC	-	169	38.5 (13.94)	87	82	36 weeks	36 weeks
CHRONOS/NCT02260986	3	AD	double	randomized	Placebo		SC	TCS	315	36.6 (13.01)	122	193	16 weeks	16 weeks
					Dupilumab	300mg q2w	SC	TCS	106	39.6 (13.98)	44	62	16 weeks	16 weeks
					Dupilumab	300mg qw	SC	TCS	319	36.9 (13.67)	128	191	16 weeks	16 weeks
NCT01859988	2	AD	double	randomized	Dupilumab	300 mg qw	SC	-	63	36.2 (10.74)	20	43	16 weeks	16 weeks
					Dupilumab	300 mg q2w	SC	-	64	39.4 (12.06)	23	41	16 weeks	16 weeks
					Dupilumab	200 mg q2w	SC	-	62	35.8 (14.90)	25	36	16 weeks	16 weeks
					Dupilumab	300 mg q4w	SC	-	65	36.8 (10.77)	25	40	16 weeks	16 weeks
					Dupilumab	100 mg q4w	SC	-	65	36.6 (11.55)	31	34	16 weeks	16 weeks
					Placebo		SC	-	61	37.2 (13.10)	21	40	16 weeks	16 weeks
NCT01548404	2	AD	double	randomized	Placebo		SC	-	54	39.4 (12.29)	27	27	12 weeks	12 weeks
					Dupilumab	300 mg	SC	-	55	33.7 (10.41)	24	31	12 weeks	12 weeks
NCT03359356	2	AA	double	randomized	Placebo		SC	-	20	46.5 (14.4)	13	7	24 weeks	24 weeks
					Dupilumab	300 mg	SC	-	40	41.6 (13.8)	30	10	24 weeks	24 weeks

UNCOVER-3/<br>NCT01646177	3	PSO	double	randomized	Placebo		SC	-	193	<=18: 0<br>18-65:<br>180<br>>=65: 13	56	137	12 weeks	12 weeks
					Etanercep<br>t	50mg	SC	-	382	<=18: 0<br>18-65:<br>349<br>>=65: 33	11<br>3	269	12 weeks	12 weeks
					Ixekizuma<br>b	80mg q4w	SC	-	386	<=18: 0<br>18-65:<br>358<br>>=65: 28	12<br>8	258	12 weeks	12 weeks
					Ixekizuma<br>b	80mg q2w	SC	-	385	<=18: 0<br>18-65:<br>351<br>>=65: 34	13<br>1	254	12 weeks	12 weeks
NCT00581555	4	PSO	double	randomized	Placebo		SC	-	62	41.50<br>(12.97)	17	45	24 weeks	24 weeks
					Etanercep<br>t	50mg	SC	-	58	41.78<br>(9.86)	20	38	24 weeks	24 weeks
NCT00078819	3	PSO	double	randomized	Placebo		SC	-	105	4-11: 38<br>12-17:<br>67	52	53	12 weeks	12 weeks
					Etanercep<br>t	0.8 mg/kg	SC	-	106	4-11:38<br>12-17:68	51	55	12 weeks	12 weeks
NCT01690299	3b	PSO	double	randomized	Placebo			-	84	43.4<br>(14.91)	25	59	16 weeks	16 weeks
					Apremilas<br>t	30mg	oral	-	83	46.0<br>(13.59)	34	49	16 weeks	16 weeks
					Etanercep<br>t	50mg	SC	-	83	47.0<br>(14.07)	34	49	16 weeks	16 weeks
UNCOVER<br>2/NCT01597245	3	PSO	double	randomized	Placebo			-	168	<=18: 0<br>18-65:<br>159<br>>=65: 9	48	120	12 weeks	12 weeks

					Etanercept	50mg	SC	-	358	<=18: 0 18-65: 337 >=65: 21	122	236	12 weeks	12 weeks
					Ixekizumab	160mg q4w	SC	-	347	<=18: 0 18-65: 324 >=65: 23	103	244	12 weeks	12 weeks
					Ixekizumab	160mg q2w	SC	-	351	<=18: 0 18-65: 327 >=65: 24	130	221	12 weeks	12 weeks
UNCOVER 1/NCT01474512	3	PSO	double	randomized	Placebo			-	431	46.4 (13.40)	128	303	12 weeks	12 weeks
					Ixekizumab	160 q4w	SC	-	432	45.6 (12.95)	143	289	12 weeks	12 weeks
					Ixekizumab	160 q2w	SC	-	433	45.1 (12.40)	142	291	12 weeks	12 weeks
NCT03364309	3	PSO	double	randomized	Placebo		SC	-	88	41.9 (12.38)	24	64	12 weeks	12 weeks
					Ixekizumab	160mg q4w	SC	-	174	41.2 (11.59)	45	129	12 weeks	12 weeks
					Ixekizumab	160mg q2w	SC	-	176	39.2 (10.53)	34	142	12 weeks	12 weeks
IXORA-PEDS/NCT03073200	3	PSO	double	randomized	Placebo		SC	-	56	13.1 (2.79)	36	20	12 weeks	12 weeks
					Ixekizumab	>50 kg: 160 mg 25-50kg: 80mg >25kg: 40mg	SC	-	115	13.7 (3.14)	63	52	12 weeks	12 weeks
NCT02008890	3	PSO	double	randomized	Secukinumab	150mg	SC	-	80	50.7 (13.68)	63	17	16 weeks	16 weeks
					Secukinumab	300mg	SC	-	79	50.6 (14.77)	64	15	16 weeks	16 weeks

					Placebo		SC	-	78	52.9 (11.33)	59	19	16 weeks	16 weeks
NCT00805480	2	PSO	double	randomized	Secukinumab	3mg/kg	SC	-	30	45.7 (9.29)	6	24	12 weeks	12 weeks
					Secukinumab	10mg/kg	SC	-	29	43.5 (11.75)	7	22	12 weeks	12 weeks
					Secukinumab	10mg/kg x 3	SC	-	31	43.4 (10.90)	5	26	12 weeks	12 weeks
					Placebo		SC	-	10	40.1 (14.89)	2	8	12 weeks	12 weeks
ALLURE/NCT02748863	3	PSO	double	randomized	Secukinumab	300mg in 2ml	SC	-	72	<65 y: 68 >=65 y: 4 >=75 y: 0	28	44	12 weeks	12 weeks
					Secukinumab	300mg in 2x 1ml	SC	-	71	<65 y: 64 >=65 y: 7 >=75 y: 0	28	43	12 weeks	12 weeks
					Placebo		SC	-	71	<65 y: 68 >=65 y: 3 >=75 y: 0	25	46	12 weeks	12 weeks
NCT03384745	2b	PSO	double	randomized	Sonelokimab	30mg	SC	-	52	48.2 (13.4)	36	16	12 weeks	12 weeks
					Sonelokimab	60mg	SC	-	52	46.9 (12.3)	38	14	12 weeks	12 weeks
					Sonelokimab	120mg q8w	SC	-	53	44.1 (13.1)	43	10	12 weeks	12 weeks
					Sonelokimab	120mg q4w	SC	-	51	43.2 (13.2)	34	17	12 weeks	12 weeks
					Placebo		SC	-	52	45.9 (12.9)	39	13	12 weeks	12 weeks
					Secukinumab	300mg	SC	-	53	47.5 (13.8)	38	15	12 weeks	12 weeks

ERASURE/NCT01365455	3	PSO	double	randomized	Secukinumab	150mg	SC	-	245	44.9 (13.33)	77	168	12 weeks	12 weeks
					Secukinumab	300mg	SC	-	245	44.9 (13.46)	76	169	12 weeks	12 weeks
					Placebo			-	248	45.4 (12.63)	76	172	12 weeks	12 weeks
SUNRISE/NCT03713632	3	HS	double	randomized	Secukinumab	300mg q2w	SC	-	181	37.3 (11.48)	98	82	16 weeks	16 weeks
					Secukinumab	300mg q4w	SC	-	180	35.5 (11.41)	103	77	16 weeks	16 weeks
					Placebo		SC	-	183	36.2 (11.25)	105	78	16 weeks	16 weeks
SUNSHINE/NCT03713619	3	HS	double	randomized	Secukinumab	300mg q2w	SC	-	182	37.1 (12.53)	102	79	16 weeks	16 weeks
					Secukinumab	300mg q4w	SC	-	181	35.7 (11.71)	100	80	16 weeks	16 weeks
					Placebo		SC	-	181	35.5 (10.75)	102	78	16 weeks	16 weeks
JUNCTURE/NCT01636687	3	PSO	double	randomized	Secukinumab	150mg	SC	-	61	43.9 (14.41)	20	41	12 weeks	12 weeks
					Secukinumab	300mg	SC	-	60	46.6 (14.23)	14	46	12 weeks	12 weeks
					placebo		SC	-	61	43.7 (12.74)	23	38	12 weeks	12 weeks
FEATURE/NCT01555125	3	PSO	double	randomized	Secukinumab	150mg	SC	-	59	46 (15.09)	19	40	12 weeks	12 weeks
					Secukinumab	300mg	SC	-	59	45.1 (12.57)	21	38	12 weeks	12 weeks
					placebo		SC	-	59	46.5 (14.14)	20	39	12 weeks	12 weeks
NCT00941031	2	PSO	double	randomized	Secukinumab	150mg qw	SC	-	66	42.7 (11.32)	13	53	12 weeks	12 weeks
					Secukinumab	150mg q4w	SC	-	138	44.2 (12.96)	34	104	12 weeks	12 weeks

					Secukinumab	150 week 1,2,3,5	SC	-	133	44.5 (12.45)	28	105	12 weeks	12 weeks
					Placebo		SC	-	67	44.2 (12.59)	23	44	12 weeks	12 weeks
NCT03066609	3	PSO	double	randomized	Secukinumab	150mg	SC	-	136	41 (11.39)	37	99	12 weeks	12 weeks
					Secukinumab	300mg	SC	-	272	39.9 (12.35)	67	205	12 weeks	12 weeks
					Placebo		SC	-	135	40.1 (11.01)	27	108	12 weeks	12 weeks
pINPOINt/NCT04237116	3	PSO	double	randomized	Secukinumab	300mg	SC	-	7	41.6 (11.8)	4	3	12 weeks	12 weeks
					Placebo		SC	-	3	32.0 (16.8)	0	3	12 weeks	12 weeks
VIP-U/NCT02187172	4	PSO	double	randomized	Ustekinumab	<100kg: 45mg >100kg: 90 mg	SC	-	22	39.45 (13.6)	6	16	12 weeks	12 weeks
					Placebo		SC	-	21	45.33 (12.76)	7	14	12 weeks	12 weeks
NCT01945086	2	AD	double	randomized	Placebo		SC	TCS	27	32.6 (10.05)	8	19	12 weeks	12 weeks
					Ustekinumab	45mg	SC	TCS	24	37.5 (8.77)	7	17	12 weeks	12 weeks
					Ustekinumab	90mg	SC	TCS	28	33.0 (8.85)	9	19	12 weeks	12 weeks
BE VIVID/NCT03370133	3	PSO	double	randomized	Placebo		SC	-	83	49.7 (13.6)	23	60	16 weeks	16 weeks
					Bimekizumab	320 mg q4w	SC	-	321	45.2 (14.0)	92	229	16 weeks	16 weeks
					Ustekinumab	45mg or 90mg	SC	-	163	46.0 (13.6)	46	117	16 weeks	16 weeks
UltIMMa-2/NCT02684357	3	PSO	double	randomized	Placebo		SC	-	98	46.3 (13.26)	31	67	16 weeks	16 weeks
					Ustekinumab	40mg or 90mg	SC	-	99	48.6 (14.81)	33	66	16 weeks	16 weeks

					Risankizumab	150mg	SC	-	294	46.2 (13.68)	91	203	16 weeks	16 weeks
UltIMMa-1/NCT02684370	3	PSO	double	randomized	Placebo		SC	-	102	49.3 (13.63)	23	79	16 weeks	16 weeks
					Ustekinumab	40mg or 90mg	SC	-	100	46.5 (13.42)	30	70	16 weeks	16 weeks
					Risankizumab	150mg	SC	-	304	48.3 (13.39)	92	212	16 weeks	16 weeks
CADMUS/NCT01090427	3	PSO	double	randomized	Placebo		SC	-	37	15.6 (1.46)	17	20	12 weeks	12 weeks
					Ustekinumab	0.375 mg/kg ≤ 60kg 22.5 mg >60kg ≤ 100kg 45mg > 100kg	SC	-	37	15.1 (1.7)	19	18	12 weeks	12 weeks
					Ustekinumab	0.75 mg/kg ≤ 60kg 45 mg >60kg ≤ 100kg 90 mg > 100kg	SC	-	36	14.8 (1.73)	20	16	12 weeks	12 weeks
PHOENIX 1/NCT00267969	3	PSO	double	randomized	Placebo		SC	-	255	44.8 (11.32)	72	183	12 weeks	12 weeks
					Ustekinumab	45mg	SC	-	255	44.8 (12.48)	80	175	12 weeks	12 weeks
					Ustekinumab	90mg	SC	-	255	46.2 (11.27)	83	173	12 weeks	12 weeks
PHOENIX 2/NCT00307437	3	PSO	double	randomized	Placebo		SC	-	410	47.0 (12.47)	127	283	12 weeks	12 weeks
					Ustekinumab	45mg	SC	-	409	45.1 (12.06)	126	283	12 weeks	12 weeks
					Ustekinumab	90mg	SC	-	411	46.6 (12.14)	137	274	12 weeks	12 weeks

AMAGINE-2/NCT01708603	3	PSO	double	randomized	Brodalumab	210mg	SC	-	612	44.5 (12.7)	191	421	12 weeks	12 weeks
					Brodalumab	140mg	SC	-	610	44.8 (12.8)	197	413	12 weeks	12 weeks
					Ustekinumab	45mg or 90 mg	SC	-	300	45.4 (13.0)	95	205	12 weeks	12 weeks
					Placebo		SC	-	309	43.7 (12.9)	90	219	12 weeks	12 weeks
BE READY/NCT03410992	3	PSO	double	randomized	Placebo		SC	-	86	43.5 (13.1)	28	58	16 weeks	16 weeks
					Bimekizumab	320mg q4w	SC	-	349	44.5 (12.9)	94	255	16 weeks	16 weeks
OASIS 2/NCT03535194	3	PSO	double	randomized	Placebo		SC	-	112	<65: 103 ≥ 65: 9	30	82	12 weeks	12 weeks
					Secukinumab	300mg	SC	-	448	<65: 399 ≥ 65: 49	137	311	12 weeks	12 weeks
CARIMA/NCT02559622	3	PSO	double	randomized	Secukinumab	300mg	SC	-	48	44.2 (12.9)	11	37	12 weeks	12 weeks
					Secukinumab	150mg	SC	-	54	46.0 (14.4)	23	31	12 weeks	12 weeks
					Placebo		SC	-	26	43.7 (11.4)	8	18	12 weeks	12 weeks
NCT01107457	2	PSO	double	randomized	Placebo		SC		27	45 (12.76)	13	14	12 weeks	12 weeks
					Ixekizumab	10mg	SC		28	47.65 (11.20)	12	16	12 weeks	12 weeks
					Ixekizumab	25mg	SC		30	45.93 (14.53)	12	18	12 weeks	12 weeks
					Ixekizumab	75mg	SC		29	46.37 (12.50)	10	19	12 weeks	12 weeks
					Ixekizumab	150mg	SC		28	45.97 (13.00)	14	14	12 weeks	12 weeks
IXORA-R/NCT03573323	4	PSO	double	randomized	Ixekizumab	160mg	SC		519	49.0 (13.90)	182	338	12 weeks	12 weeks

					Guselkumab	100mg	SC	506	49.0 (14.88)	193	314	12 weeks	12 weeks
NCT01806662	2	AD	double	randomized	Ustekinumab		SC	16	38.5 (18 to 61)	12	20	16 weeks	16 weeks
					Placebo		SC	16	38.5 (18 to 61)	12	20	16 weeks	16 weeks

Table S4 – Risk of bias assessment

	Randomization	allocation - assignment	Missing outcomes data	outcomes measures	reporting bias	Overall risk of bias
NCT02201524	LOW	LOW	LOW	LOW	LOW	LOW
NCT02576938	low	LOW	LOW	LOW	LOW	LOW
NCT02780167	LOW	LOW	LOW	LOW	LOW	LOW
NCT02925117	LOW	LOW	LOW	LOW	LOW	LOW
BREEZE-AD2/NCT03334422	LOW	LOW	LOW	LOW	LOW	LOW
BREEZE-AD4/NCT03428100	LOW	LOW	LOW	LOW	LOW	LOW
AD Up/ NCT03568318	LOW	LOW	LOW	LOW	LOW	LOW
Measure up 1/ NCT03569293	LOW	LOW	LOW	LOW	LOW	LOW
BRAVE-AA1/NCT03570749	LOW	LOW	LOW	LOW	LOW	LOW
JADE MONO/NCT03575871	LOW	LOW	LOW	LOW	LOW	LOW
Measure up 2/ NCT03607422	LOW	LOW	LOW	LOW	LOW	LOW
JADE COMPARE/NCT03720470	LOW	LOW	HIGH	LOW	LOW	HIGH
Heads up/ NCT03738397	LOW	LOW	LOW	LOW	LOW	LOW
BRAVE-AA2/ NCT03899259	LOW	LOW	LOW	LOW	LOW	LOW
Jade MOA/NCT03915496	LOW	LOW	LOW	LOW	LOW	LOW
Jade MOA/NCT03915496	LOW	LOW	LOW	LOW	LOW	LOW
Jade DARE/NCT04345367	LOW	LOW	LOW	LOW	LOW	LOW
NCT04822584	LOW	LOW	LOW	LOW	LOW	LOW

NCT05556265	LOW	LOW	LOW	LOW	LOW	LOW
Measure up 1/ NCT03569293	LOW	LOW	LOW	LOW	LOW	LOW
Measure up 2/ NCT03607422	LOW	LOW	LOW	LOW	LOW	LOW
PIONEER I/NCT01468207	LOW	LOW	LOW	LOW	LOW	LOW
PIONEER II/NCT01468233	LOW	LOW	LOW	LOW	LOW	LOW
NCT00918255	LOW	LOW	LOW	LOW	LOW	LOW
NCT01251614	LOW	LOW	UNCLEAR	LOW	LOW	UNCLEAR
NCT03248531	LOW	LOW	UNCLEAR	LOW	LOW	UNCLEAR
VOYAGE 1/NCT02207231	LOW	LOW	LOW	LOW	LOW	LOW
VOYAGE 2/NCT02207244	LOW	LOW	LOW	LOW	LOW	LOW
NCT02694523	LOW	LOW	LOW	LOW	LOW	LOW
JADE COMPARE/NCT03720470	LOW	LOW	LOW	LOW	LOW	LOW
Heads up/ NCT03738397	LOW	LOW	LOW	LOW	LOW	LOW
NCT04678882	LOW	LOW	LOW	LOW	LOW	LOW
NCT04345367	LOW	LOW	LOW	LOW	LOW	LOW
SOLO-1/NCT02277743	LOW	LOW	UNCLEAR	LOW	LOW	UNCLEAR
SOLO 2/NCT02277769	LOW	LOW	UNCLEAR	LOW	LOW	UNCLEAR
SOLO-CONTINUE/NCT02395133	LOW	LOW	LOW	LOW	LOW	LOW
CHRONOS/NCT02260986	LOW	LOW	LOW	LOW	LOW	LOW
NCT01859988	LOW	LOW	LOW	LOW	LOW	LOW
NCT01548404	LOW	LOW	LOW	LOW	LOW	LOW
NCT03359356	LOW	LOW	LOW	LOW	LOW	LOW
UNCOVER-3/NCT01646177	LOW	LOW	LOW	LOW	LOW	LOW
NCT00581555	LOW	LOW	LOW	LOW	LOW	LOW
NCT00078819	LOW	LOW	LOW	LOW	LOW	LOW
NCT01690299	LOW	LOW	LOW	LOW	LOW	LOW
UNCOVER 2/NCT01597245	LOW	LOW	LOW	LOW	LOW	LOW
UNCOVER 1/NCT01474512	LOW	LOW	LOW	LOW	LOW	LOW
NCT03364309	LOW	LOW	LOW	LOW	LOW	LOW
NCT02008890	LOW	LOW	LOW	LOW	LOW	LOW
NCT00805480	LOW	LOW	LOW	LOW	LOW	LOW

ALLURE/NCT02748863	LOW	LOW	LOW	LOW	LOW	LOW
NCT03384745	LOW	LOW	LOW	LOW	LOW	LOW
ERASURE/NCT01365455	LOW	LOW	LOW	LOW	LOW	LOW
SUNRISE/NCT03713632	LOW	LOW	LOW	LOW	LOW	LOW
SUNSHINE/NCT03713619	LOW	LOW	LOW	LOW	LOW	LOW
JUNCTURE/NCT01636687	LOW	LOW	LOW	LOW	LOW	LOW
FEATURE/NCT01555125	LOW	LOW	LOW	LOW	LOW	LOW
NCT00941031	LOW	LOW	LOW	LOW	LOW	LOW
NCT03066609	LOW	LOW	LOW	LOW	LOW	LOW
pINPOINt/NCT04237116	LOW	LOW	LOW	LOW	LOW	LOW
VIP-U/NCT02187172	LOW	LOW	LOW	LOW	LOW	LOW
NCT01945086	LOW	LOW	LOW	LOW	LOW	LOW
BE VIVID/NCT03370133	LOW	LOW	LOW	LOW	LOW	LOW
UltIMMa-2/NCT02684357	LOW	LOW	LOW	LOW	LOW	LOW
UltIMMa-1/NCT02684370	LOW	LOW	LOW	LOW	LOW	LOW
CADMUS/NCT01090427	LOW	LOW	LOW	LOW	LOW	LOW
PHOENIX 1/NCT00267969	LOW	LOW	LOW	LOW	LOW	LOW
PHOENIX 2/NCT00307437	LOW	LOW	LOW	LOW	LOW	LOW
AMAGINE-2/NCT01708603	LOW	LOW	LOW	LOW	LOW	LOW
BE READY/NCT03410992	LOW	LOW	LOW	LOW	LOW	LOW
OASIS 2/NCT03535194	LOW	LOW	LOW	LOW	LOW	LOW
CARIMA/NCT02559622	LOW	LOW	LOW	LOW	LOW	LOW
NCT01107457	LOW	LOW	LOW	LOW	LOW	LOW
IXORA-R/NCT03573323	LOW	LOW	LOW	LOW	LOW	LOW
NCT01806662	LOW	LOW	LOW	LOW	LOW	LOW
NCT02201524	LOW	LOW	LOW	LOW	LOW	LOW
NCT01490632	LOW	LOW	LOW	LOW	LOW	LOW
NCT01815424	LOW	LOW	LOW	LOW	LOW	LOW
NCT01309737	LOW	LOW	HIGH	LOW	LOW	HIGH
